# Current practices in robotic surgery for distal ureter in children: results from an EAU robotic urology section (ERUS) survey

**DOI:** 10.3389/fped.2026.1729840

**Published:** 2026-02-10

**Authors:** Girolamo Mattioli, Luca Genova Gaia, Marcello Carlucci, Andrea Minervini, Furkan Sendogan, Mesrur Selcuk Silay, Hubert John, Venusia Fiorenza

**Affiliations:** 1 DINOGMI, University of Genoa, Genoa, Italy; 2 Pediatric Surgery Unit, IRCCS Istituto Giannina Gaslini, Genoa, Italy; 3 Department of Urology, University of Florence, Careggi Hospital, Florence, Italy; 4 Pediatric Urology Unit, Medipol Acibadem Hospital, Clinic of Pediatric Urology, Istanbul Medipol University, Istanbul, Türkiye; 5 Department of Urology, Kantonsspital Winterthur, Winterthur, Switzerland

**Keywords:** pediatric urology, robotic surgery, ureteral reconstruction, ureteral reimplantation, ureteral surgery

## Abstract

**Introduction:**

The robotic approach for uretero-vesical junction (UVJ) anomalies in children is still limited and debated. Both paediatric and adult urologists perform these procedures. This survey-based descriptive study aimed to evaluate different robotic approaches to the UVJ anomalies and their spread among ERUS Urologists performing robotic surgery in children, focusing on indications, technical aspects and outcomes.

**Materials and methods:**

A survey was distributed to ERUS members to gather data on paediatric patients treated for UVJ anomalies between January 2017 and December 2022. Data were collected on demographics, diagnoses, surgical details, complications, and postoperative outcomes.

**Results:**

Three Centres participated in the survey. A total of 153 patients were included in the study. Centre 1 participated with a series of 6 patients: (4 D-RALUR, ND-RALUR). Centre 2 participated with a series of 67 patients (2 D-RALUR, 65 ND-RALUR). Centre 3 participated with a series of 80 patients (56 D-RALUR, 16 ND-RALUR and 7 UU). The surgery success rate varied from 50% to 100%. No life-threatening events were reported. The main complications were the need for further surgery due to persistent VUR or post-operative UVJ obstruction.

**Conclusions:**

Robotic approach to the distal ureter in children is still not widely performed by ERUS Urologists. The most performed procedure is ND-RALUR. Robotic-assisted distal ureter surgery seems to be safe and feasible in paediatric patients, but further studies and experience are needed. This survey provides a general overview of the robotic-assisted surgical approach to distal ureter disease in the paediatric population and its different popularity and outcomes.

## 1 Introduction

Interest in pediatric robotic surgery has grown in recent years. Urology seems to be particularly benefiting from this technology, as seen in adult cases (1). Indeed, robotic technology improves surgeons' vision, movements and precision (2) especially in limited anatomical spaces.

Improved safety and efficacy in pediatric cases have been reported, promoting surgeons to expand indications for robot-assisted procedures (3–5). While surgery for ureteropelvic junction (UPJ) in adults has been widely performed for years and supported by scientific evidence (6, 7), the approach to the ureterovesical junction (UVJ) and distal ureter in pediatric patients remains less common and less standardized.

Pediatric UVJ pathologies include vesicoureteral reflux (VUR), primary obstructive megaureter (POM), refluxing-obstructive megaureter (ROM) and secondary megaureter (sec MU). Several surgical techniques have been reported. The most popular procedure is the open cross trigonal reimplantation by Cohen, which has favourable outcomes but is associated with important limitations. Minimally invasive procedures are still less common but seem to have some advantages and the EAU guidelines do not yet recommend these approaches as a routine technique (6). Robot-assisted laparoscopic ureteral reimplantation (RALUR) is suggested in patients with complex anatomy and/or after failed injective treatment or open ureter reimplantation.

The aim of this survey is to evaluate different robotic approaches for UVJ anomalies among ERUS urologists who perform pediatric robotic surgery, focusing on indications, technique variations, short and long-term outcomes.

## 2 Materials and methods

Between December 2022 and March 2023, ERUS members were invited by the leading Centre to participate in this survey. Respondents were asked to complete a questionnaire on pediatric patients treated between January 2017 and December 2022 for UVJ anomalies with intravesical robotic vesicoureteral reimplantation, extravesical robotic dismembered reimplantation (D-RALUR), extravesical robotic non-dismembered reimplantation (ND-RALUR) and uretero-ureterostomy (UU). Patients up to 18 years of age, who underwent robotic-assisted surgical treatment for VUR, POM, ROM, or sec MU and who had not received previous treatment or had not responded to conservative, endoscopic, or surgical interventions were included. Patients were excluded if they were over 18 years old, had a follow-up period of less than six months, or had a neurological bladder.

The questionnaire was structured in three sections, as shown in Appendix 1. The first section contained demographic data on patients and clinical indications for the procedure performed. The second section concerned surgical details of the procedure. Finally, the last section was dedicated to information about follow-up and outcomes.

The data to be entered and the questions for the centres were devised by the paediatric urology team at the leading centre based on the main points of interest in the literature on this subject.

Demographics, diagnosis, surgical details, complications and post-operative outcomes were recorded by participating centres and collected by the leading centre. Collected data were analysed with descriptive statistics.

As regards the definition of surgical success, clinical success was defined as resolution of symptoms, while radiological success was defined as evidence from instrumental examinations of resolution of the obstructive or reflux condition.

### 2.1 Surgical techniques

The robotic techniques assessed included D-RALUR, ND-RALUR, and UU. We collected details of patient positioning, port placement, ureteral isolation, and reimplantation approaches as applied by the surgeons at each participating centre. Specific surgical steps varied slightly among centres based on procedural preferences and institutional protocols.

D-RALUR: chosen in patients with UVJ primary or secondary obstruction and with ROM.

Patient supine, 15–20° Trendelenburg position. A bladder catheter is placed. The 8 mm 0° camera port is placed at the umbilical site. Two or three 8 mm ports are placed on the transverse umbilical line, with a minimal distance of 4 cm between ports. The ureter is identified at or below its crossing point with the iliac vessels and mobilized. The UVJ is exposed and isolated, preserving its vascular peduncles, vas deferens and uterine artery. Detrusotomy is then performed without mucosa layer opening. The distal part of the ureter is resected at the UVJ. If not placed before the procedure, the ureteral stent is inserted. If required, ureteral remodelling is performed according to the surgeon's preferences. The anastomosis is completed and the detrusor is sutured, wrapping the ureter. In case of tense anastomosis, the bladder dome could be mobilized to better reach the ureter or a psoas hitch can be performed.

ND-RALUR: usually performed in patients with VUR without signs of obstruction. Patient positioning, trocar placement and ureteral isolation and dissection are similar to D-RALUR. The ureter is not resected but directly reimplanted over the bladder mucosal layer, according to the Lich-Gregoir technique.

UU: considered for patients affected by a duplex collecting system with obstruction or ectopy of the upper moiety ureter or a single system with ureteral stricture and short ureters, usually secondary to previous surgeries at the UVJ; it can be performed by ipsilateral or contralateral anastomosis.

In cases of duplex systems, an ipsilateral UU is performed. The orthotopic non-refluxing ureter is stented by preoperative cystoscopy. Trocars placement is the same as for RALUR. The ureters are identified below their crossing with the iliac vessels. The pathological one is then mobilised, ligated, and resected as distally as possible. An ureterotomy of approximately 1 cm in length is performed on the orthotopic ureter and an end-to-side anastomosis is completed.

In cases of single systems with long-standing obstruction and/or ischemia of the distal ureter, especially in those patients who have previously undergone surgical treatments that may result in insufficient ureter length, a crossed UU could be performed. In this case, as well, a cystoscopy is performed prior to the robotic procedures to stent the healthy ureter on the contralateral side. The ureter to be reimplanted is isolated and dissected as distally as possible and the pathological distal tract is removed. The contralateral ureter can be reached through a retrocolic window. Indocyanine green fluorescence (ICG) could be used to check the ureteral blood supply. Ureterotomy is performed on the contralateral ureter and the end-to-side anastomosis is completed.

## 3 Results

Three Centres participated in the survey: IRCCS G. Gaslini, Genoa (Italy), AOU Careggi, Florence (Italy) and Memorial Hospital Group, Istanbul (Turkey).

A total of 153 patients were included (6 Florence, 67 Istanbul, 80 Genoa).

Surgical procedures performed were ND-RALUR in 84 patients, D-RALUR in 62 and UU in 7. Main results are summarised in Tables 1–3.

**Table 1:** Summary of ERUS survey results—part 1.

Centre and procedure	Patients (N°)	Mean age (years)	Mean weight (KG)	Mean operative time (minutes)	Number of trocar	Submucosal tunnel	Remodelling technique (N°)
CENTRE 1	**6**						
D-RALUR	4	7.7	27	219	3	Ureter diameter×3	Hendren (1)
ND-RALUR	2	2.1	14	124	3	Ureter diameter×3	
UU	0	—	-	—		—	
CENTRE 2	**67**						
D-RALUR	2	1.1	10.5	155	3		Hendren (2)
ND-RALUR	65	4.5	25.8	123	3	1:5 ureter diameter/intramural length	
UU	—	—	—	-	—	—	
CENTRE 3	**80**						
D-RALUR	56	3.9	17.1	166	3 or 4	4.1 cm (range 3–6)	Hendren (11) Starr (6) Kalicinski (1)
ND-RALUR	17	4.3	15.5	122	3 or 4	4 cm (range 2–4)	
UU	7	5.4	24.4	144		—	

D-RALUR, dismembered robotic-assisted laparoscopic ureteral reimplantation; ND-RALUR, non-dismembered robotic-assisted laparoscopic ureteral reimplantation; UU, ureterouretrostomy.

The total number of patients for each center is highlighted in bold.

**Table 2:** Summary of ERUS survey results—part 2.

Centre and procedure	Patients (N°)	Diagnosis	Previous treatments	Ureteral stent	Stent time (DAYS)	Bladder catheter time (mean days)	Length of stay (mean days)
CENTRE 1	**6**						
D-RALUR	4	POM	0	INTRA-OP	30–90	5	6
ND-RALUR	2	VUR	1	NO	—	1	2.5
UU	0	—	-	—	-	—	-
CENTRE 2	**67**						
D-RALUR	2	ROM	0	INTRA-OP	30–90	3	3
ND-RALUR	65	VUR	14	NO/PRE-OP	14	0.9	1.7
UU	—	—	—	—	—	—	—
CENTRE 3	**80**						
D-RALUR	56	VUR (21) Sec MU (13) POM (13) ROM (9)	25	INTRA-OP	30–90	1.15	3.6
ND-RALUR	17	VUR	8	NO	—	2	4.9
UU	7	POM (4)					
Sec MU (3)	3	PRE-OP	30–90	1.8	3.4		

The total number of patients for each center is highlighted in bold.

**Table 3:** Summary of ERUS survey results—part 3.

Centre and procedure	Patients (N°)	Resolution rate (%)	Post-operative VUR (%)	Post-operative UVJ obstruction (%)	Follow up (mean months)
CENTRE 1	**6**				
D-RALUR	4	**50**	50	—	44
ND-RALUR	2	**50**	50	—	12
UU	0	—	—	—	-
CENTRE 2	**67**				
D-RALUR	2	**100**	—	—	21
ND-RALUR	65	**97**	—	—	20.2
UU	—	—	—	—	—
CENTRE 3	**80**				
D-RALUR	56	**67**	27	6	16
ND-RALUR	17	**71**	—	29	20.7
UU	7	**86**	—	14	17.5

UVJ, uretero-vesical junction.

The total number of patients for each center is highlighted in bold.

Among D-RALUR, mean operative time range was between 155 and 219 min, while in ND-RALUR was between 122 and 124 min. The most reported ureteral remodelling technique reported was the one according to Hendren. All centres reported using different methodologies for planning the submucosal tunnel for ureterovesical reimplantation. The indication for ND RALUR was the same for all centres, i.e., vesicoureteral reflux, while for D-RALUR the indications were more varied and less uniform between the enrolled Centres.

All the Centres reported the clinical success for each procedure. Only one Centre recorded both clinical and radiological success.

Complications classified as IIIb according to Clavien Dindo classification (7) were collected specifically. Need for endoscopic treatment after surgery were considered as grade IIIb as well, due to need for general anesthesia. The main results concerning D-RALUR and ND-RALUR are summarised in Table 4.

**Table 4:** Summary of ERUS survey overall results—All centres combined.

Overall results	ND-Ralur	D-Ralur
Patients (n)	84	62
Age (years, range)	0.5–17.1	0.6–17.2
Weight (kg, range)	8.0–135.0	7.2–80.0
Operative time (min, range)	60–230	93–305
Diagnosis	VUR	VUR (21) POM (17) ROM (11) Sec MU (13)
Stent removal timing (days, range)	14	30–90
Bladder Catheter (mean days range)	0.9–2	1.1–5
Length of stay (mean days, range)	1.7–4.9	3–6
Resolution (% range)	50–97	50–100
Post-OP VUR (% range)	0–50	0–50
Post-OP UVJ obstruction (% range)	0–29	0–6
Follow-up (mean months, range)	12–20.7	16–44

No intraoperative complications and no conversion to open procedure were reported by the centres.

Centre 1 (Florence) used DaVinci Si in ND-RALURs and DaVinci Xi in D-RALURs. Centre 2 (Istanbul) and Centre 3 (Genoa) used DaVinci Xi.

## 4 Discussion

In recent years, the adoption of robot-assisted surgery in pediatric settings has increased, especially in urology (4, 8). Cohen's open reimplantation remains the most performed operation with excellent results reported in the literature (9, 10). As new minimally invasive surgical techniques have become available, numerous authors have analyzed and compared the different approaches in terms of feasibility, cost, safety, and outcomes (11–14).

An important issue in the patient undergoing Cohen's reimplantation is the frequent difficulty in performing endoscopic maneuvers such as stent placement or ureterorenoscopy (15, 16). Other issues are pain, bladder spasm, length of stay, need for drain tubes, and risk of vas deferens injuries. All of them seem to be improved by a robotic approach (13, 17–25). Better visualization can reduce the risk of pelvic plexus and vas deferens damage (26). Urinary retention is reported as a common complication after ureteral reimplantation (27), with a frequency up to 10%, especially in bilateral cases (28, 29), but seems to be less frequent in robotic surgery compared to open procedures (27). This complication was not reported in our survey. Open procedures are thought to carry a higher risk of voiding dysfunction because of the cystotomy or because of neuropraxia from dissection (26, 30).

ND-RALUR seems to be safe and effective even in patients with previous surgical treatment and complex anatomy (31). Literature on D-RALUR remains sparse, although preliminary data and the experiences of our centres seem encouraging.

VUR is the most common indication for pediatric reimplantation. Less frequent indications are POM, sec MU, ROM, ureterocele and ectopic ureters. Even if RALUR is still not considered the gold standard procedure, it can be a valid choice in patients with complex anatomy and/or after failed treatments, as reported by EAU 2024 guidelines (6, 32).

We found a variation in procedural preferences, between ND and D-RALUR. Overall, ND-RALUR was the most performed procedure, but the prevalence varies depending on the Institution. In the two series with the higher number of cases, one centre performed almost exclusively ND-RALUR, while the other one performed both procedures with a higher number of D-RALUR. This is related to the different surgical indications depending on the underlying pathology, thus in patients' selection. Regarding Centre 2, we do not know if the lower number of D-RALUR compared to ND-RALUR is due to a lack of indications or a preference for a different surgical approach, such as open surgery, for more complex cases.

As regards the detrusor tunnelling, some authors use the diameter of the distal ureter as a parameter, with different ratios reported (28, 33, 34). Other authors use a specific length (35). Results from this survey are similarly not uniform: Centre 3 reported a mean length of 4 cm in ND-RALUR procedure and 4.1 cm in D-RALUR, while Centre 1 used the 3:1 rule. Centre 2 reported no details on this topic.

In bilateral UVJ pathology, it is important to consider whether to perform the correction in one or two stages. Some Authors reported higher risk of post-operative urinary retention and complications in bilateral procedure (36, 37). Recently Hajiyev showed that bilateral RALUR seems to be safe and not related to a higher risk of complications (38). In this ERUS survey, there was no evidence of differences in outcomes among bilateral procedures, and no cases of acute postoperative urinary retention were reported. The 3D visualization provided by robotic technology allows visualization of the neurovascular bundle and avoidance of intraoperative damage, as suggested by other Authors (38).

All three Centers usually removed the urinary catheter on the first postoperative day, similar to the literature (26, 27, 39). Longer catheterization was typically due to a suspected urinary leak. Interestingly, some Authors report no need for post-operative catheter after ND-RALUR in unilateral primary VUR (40). On the other hand, some Centres keep the catheter for a longer time after dismembered procedures (41).

In patients with severe ureter distension, a distal remodelling is frequently chosen. The most popular techniques are tapering by Hendren (42) and tailoring by Kalichinski (43) or Starr (44). This could provide a better urine flow (45) and prevent VUR, although with no strong scientific basis for effectiveness (46, 47). In our survey, the Hendren type was the most frequently performed by all Centres.

An intraluminal stent is generally used in case of D-RALUR to protect the anastomosis and prevent leak or transient obstruction (27, 48). Even if stentless pyeloplasty has been described (49–51), there is no evidence about stentless robotic ureteral reimplantation in terms of safety and efficacy. In ND procedures, stenting is usually not performed (14). This survey showed conformity between Centers 1 and 3, which did not use the stent in the ND-RALUR; Centre 2 placed the stent before performing ND-RALUR only in patients with abnormal anatomy, such as a duplex system or solitary kidney. In D-RALUR procedures, all Centers placed the stent during the robotic procedure. Some Authors prefer to place it with a preliminary cystoscopy (32).

The timing of stent removal in our D-RALUR groups seems to be slightly longer than that suggested by other Authors (41, 52), which is usually between 2 and 8 weeks. Nevertheless, there is no consensus. Interestingly, some studies about ureteric surgery showed that an early removal might be safe without increasing the risk of urinary leak (53–55).

Literature about robotic outcomes is still discordant (29, 56)**.** RALUR outcomes are quite variable, with a surgical success rate between 77% and 100% (28). In ND-RALUR, persistent VUR rate is 23% (29). The overall complication rate for ND-RALUR is variable among the Authors' reports, with a wide range (0%–14%) (26, 57–59). One of the most important complications is ureteral obstruction, usually due to transient oedema, thus suggesting that stent placement is mandatory in case of a solitary kidney or persisting obstruction (27). The need for postoperative stenting ranges between 0% and 10% (25, 60, 61). In this survey, 5 cases treated by ND-RALUR required a post-operative ureteral stenting due to UVJ obstruction, describing a post-operative obstruction rate of 29.4%. Stenting was effective in solving the obstruction.

After D-RALUR, anastomotic leak and stenosis are the most concerning complications. Centre 3 reported 4 patients with post-operative UVJ obstruction (7.1%) treated by endoscopic stenting. After an open procedure, 6.6% of patients experience post-operative obstruction (62). Literature on obstruction after dismembered reimplantation is still poor; Mittal reported no leak or stenosis after D-RALUR for POM, finding a general low complication rate, comparable to ND-RALUR (41). Even regarding obstruction rates after D-RALUR, the results from this survey seem similar to those of other Authors' experiences (47, 52). A temporary postoperative hydronephrosis is not rare, and the resolution is usually spontaneous; this finding seems to be comparable with open and robotic reimplantation (63).

De-novo or persistent VUR is another major issue after ND procedures and it's usually defined by post-op VCUG, with variable grade and rate between Authors (29, 37). Regarding VUR after dismembered procedures, there are few data (64, 65). In general, surgical success for VUR treatment can be defined by symptoms resolution or radiologically defined as the absence or reduction of reflux. For this reason, there is often no uniformity in the literature (31). In this ERUS survey, Centre 1 and Centre 3 reported clinical success, while Centre 2 provided both clinical and radiological success. Regarding ND-RALUR group the reported success rate ranged between 50% and 100%. Centre 1 reported a 50% rate; Centre 2 reported a clinical success rate of 97% and a radiological one of 100%, but only 33% of the patients had undergone VCUG. Centre 3 reported a 71% success rate. In D-RALUR, success rates varied from 67% to 100% (67% from Centre 3, 50% from Centre 1 and 100% from Centre 2, but the last series only consists of 2 procedures).

Open techniques for VUR repair have well-known good outcomes (66, 67). Nevertheless, a recent meta-analysis suggests similar outcomes between ND-RALUR and open reimplantation in VUR patients (58), but further long-term studies are needed. RALUR complexity seems to be underestimated and its learning curve is long, which potentially may affect success rates analysis (61).

Ureteroureterostomy is mostly performed in children with mid-ureteral obstruction and minimally invasive techniques seem to be safe with good results, even compared to open surgery (68–71). The anastomosis can be ipsilateral or contralateral (trans-ureteroureterostom y^72^). In this survey, only Centre 3 performed UU and the main indication was duplex system. Similarly to data from literature, the reported success rate was 85.7% with a low complication rate (73). Only one patient among contralateral anastomosis group had post-operative obstruction, which was solved with temporary stenting. The mean operative time was similar or shorter than other series (73, 74), although the definition of procedure timing may be different depending on the Author. UU seems to be a safe and effective alternative to the same open procedure (68, 75, 76). Nevertheless, despite encouraging results, this procedure is not yet widely performed in pediatric patients.

The main limitations of our survey are the heterogeneous patient samples, the retrospective design of the study and the descriptive nature since it is a survey. Moreover, this survey did not focus on differences between surgeons' skill and experience, as it didn't analyse the respective learning curves.

## 5 Future perspectives

Given the increasing availability and uptake of robotic surgery in paediatrics, we believe that reconstructive ureteral procedures represent a suitable platform for further developing and refining these techniques. To strengthen the evidence base, more standardised and prospective studies will be required. Moreover, because paediatric surgery inherently involves smaller patient cohorts compared with adult practice, we consider multicentre and international collaboration essential to ensure adequate sample sizes and more generalisable findings.

## 6 Conclusions

This survey collected a substantial cohort of pediatric patients who underwent robotic surgery of the distal ureter. Our findings suggest that adult urologists do not frequently perform pediatric robotic procedures, which is becoming increasingly popular among pediatric urological specialists.

The main indication for RALUR among ERUS members is VUR, according to the literature.

RALUR has gained popularity, showing promising outcomes (41, 77). Although results are still not comparable to open techniques, urologists must keep on developing minimally invasive approaches, comparing results and discussing indications and procedures.

Further multicentric studies and surveys are needed to improve the development and the popularity of robotic procedures treating distal ureteral pathologies in children.

## Data Availability

The raw data supporting the conclusions of this article will be made available by the authors, without undue reservation.

## Appendix 1. ERUS Survey questionnaire details

**SECTION A—EXTRA-VESICAL ROBOTIC DISMEMBERED REIMPLANTATION (D-RALUR)**

**A1—Indications**

1. Patients treated for vesico-ureteral reflux (VUR)
2. Patients treated for obstructive megaureter
3. Patients treated for obstructive + refluxing megaureter
4. Patients treated for other indications **(**type and number)

**A2—Patient Characteristics**

5. Age at surgery (mean and range, years)
6. Weight at surgery (mean and range, kg)
7. Female patients
8. Male patients

**A3—Operative Details**

9. Unilateral operations
10. Bilateral operations
11. Duplex systems operated
12. Previous endoscopic bulking injection
13. Previous reimplantation (type and number)
14. Mean VUR grade (if applicable)
15. Robotic system used
16. Number of robotic trocars
17. Accessory laparoscopic trocar used?
18. Ureteral remodelling performed
19. Remodelling technique
20. Submucosal tunnel length (mean and range, cm)
21. Psoas hitch performed?
22. Pre-operative stent?
23. Intra-operative stent?
24. Stent removal timing

**A4—Outcomes**

25. Intra-operative complications (type and number)
26. Conversions to open surgery (number, indications)
27. Operative time (mean and range, min)
28. Follow-up duration (mean and range, months)
29. Bladder catheter duration (mean and range, days)
30. Length of hospital stay (mean and range, days)
31. VUR after surgery
32. UVJ obstruction after surgery
33. Resolved cases
34. Secondary surgeries (type and number)
35. Additional comments

**SECTION B—EXTRA-VESICAL ROBOTIC NON-DISMEMBERED REIMPLANTATION (ND-RALUR)**

**B1—Indications**

36. Patients treated for VUR
37. Patients treated for other indications (type and number)

B2—Patient Characteristics

38. Age (mean and range, years)
39. Weight (mean and range, kg)
40. Female patients
41. Male patients

B3—Operative Details

42. Unilateral operations
43. Bilateral operations
44. Duplex systems operated
45. Previous bulking injection
46. Previous reimplantation (type and number)
47. Mean VUR grade
48. Robotic system used
49. Number of robotic trocars
50. Accessory laparoscopic trocar?
51. Number of ureteral remodelling
52. Remodelling technique
53. Submucosal tunnel length (mean, range)
54. Psoas hitch performed?
55. Pre-operative stent?
56. Intra-operative stent?
57. Stent removal timing

**B4—Outcomes**

58. Intra-operative complications
59. Conversions to open surgery
60. Operative time (mean and range, min)
61. Follow-up duration (months)
62. Bladder catheter duration (days)
63. Length of hospital stay (mean and range, days)
64. VUR after surgery
65. UVJ obstruction after surgery
66. Patients considered resolved
67. Secondary surgeries (type and number)
68. Additional comments

**SECTION C—ROBOTIC URETERO-URETEROSTOMY (UU)**

**C1—Indications**

69. Ipsilateral UU for incontinence (duplex systems)
70. Ipsilateral UU for obstruction (duplex)
71. Ipsilateral UU for VUR (duplex)
72. Ipsilateral UU for obstruction + VUR (duplex)
73. Contralateral UU for incontinence (single systems)
74. Contralateral UU for obstruction (single)
75. Contralateral UU for VUR (single)
76. Contralateral UU for obstruction + VUR (single)
77. Other indications (type and number)

**C2—Patient Characteristics**

78. Age (mean and range, years)
79. Weight (mean and range, kg)
80. Female patients
81. Male patients

**C3—Operative Details**

82. Unilateral procedures
83. Bilateral procedures
84. Previous bulking injection
85. Previous reimplantation (type and number)
86. Previous UVJ surgeries (type and number)
87. Robotic system used
88. Number of robotic trocars
89. Accessory laparoscopic trocar?
90. Ureteral remodelling
91. Remodelling technique
92. Associated procedures (e.g., reimplantation) (type and number)
93. Pre-operative stent?
94. Intra-operative stent?
95. Stent removal timing

**C4—Outcomes**

96. Intra-operative complications (type and number)
97. Conversions to open surgery
98. Operative time (mean and range, min)
99. Follow-up duration (months)
100. Bladder catheter duration (days)
101. Hospital stay (days)
102. Anastomotic obstruction after surgery
103. Patients considered resolved
104. Secondary surgeries (type and number)
105. Additional comments
